# Humoral antibody response following mRNA vaccines against SARS-CoV-2 in solid organ transplant recipients; a status after a fifth and bivalent vaccine dose

**DOI:** 10.3389/fimmu.2023.1270814

**Published:** 2023-11-27

**Authors:** Emma Christophorou, Anna Christine Nilsson, Inge Petersen, Susan O. Lindvig, Jesper R. Davidsen, Rozeta Abazi, Mikael K. Poulsen, Rune M. Pedersen, Ulrik S. Justesen, Nicolai E. Johansen, Claus Bistrup, Lone W. Madsen, Isik S. Johansen

**Affiliations:** ^1^ Department of Infectious Diseases, Odense University Hospital, Odense, Denmark; ^2^ Department of Clinical Research, University of Southern Denmark, Odense, Denmark; ^3^ Department of Clinical Immunology, Odense University Hospital, Odense, Denmark; ^4^ South Danish Center for Interstitial Lung Diseases (SCILS), Department of Respiratory Medicine, Odense University Hospital, Odense, Denmark; ^5^ Department of Gastroenterology, Odense University Hospital, Odense, Denmark; ^6^ Department of Cardiology, Odense University Hospital, Odense, Denmark; ^7^ Department of Clinical Microbiology, Odense University Hospital, Odense, Denmark; ^8^ Department of Nephrology, Odense University Hospital, Odense, Denmark; ^9^ Department of Regional Health Research, University of Southern Denmark, Odense, Denmark; ^10^ Unit for Infectious Diseases, Department of Medicine, Lillebaelt Hospital, Kolding, Denmark; ^11^ Open Patient Data Explorative Network, Odense University Hospital, Odense, Denmark

**Keywords:** COVID-19 vaccination, solid organ transplant, humoral response, fifth dose, bivalent vaccines, BA.1, BA4/BA5, breakthrough infections

## Abstract

**Background:**

In solid organ transplant (SOT) recipients, the humoral response following COVID-19 vaccination is reduced, as a result of their immunosuppressed treatment. In this study, we investigated antibody concentrations after booster vaccinations until the fifth dose, the latter by monovalent or bivalent BA1 or BA4/5 vaccines. In addition, we evaluated the efficacy of vaccination by recording breakthrough infections, hospitalizations, and deaths.

**Method:**

This prospective cohort study included 438 SOT recipients (>18 years) vaccinated with mRNA vaccines against COVID-19 from January 2021 until March 2023. Blood samples were drawn before and after each vaccination and tested for SARS-CoV-2 spike RBD IgG antibodies with the lowest and highest cut-off at 7.1 and 5,680 BAU/mL, respectively. Vaccine information, breakthrough infections, and hospitalizations were collected from the medical records.

**Results:**

Most participants received BNT162b2 and 61.4% received five vaccine doses. The response proportion in SOT recipients increased from 86.7% after the fourth dose to 93.0% following the fifth dose. Antibody concentration decreased with 142.7 BAU/mL between the third and fourth dose (median 132 days, Quartile 1: 123, Quartile 3: 148) and 234.3 BAU/mL between the fourth and fifth (median 250 days, Quartile 1: 241, Quartile 3: 262) dose among those without breakthrough infection (p=0.34). When comparing the Omicron BA.1 or Omicron BA.4/BA.5 adapted vaccines, no significant differences in antibody concentration were found, but 20.0% of SOT recipients receiving a monovalent fifth vaccine dose had a breakthrough infection compared to 4.0% and 7.9% among those who received BA.1 and BA.4/BA.5 adapted vaccines, respectively (p=0.04). Since January 2021, 240 (54.8%) participants had a breakthrough infection, and 22 were hospitalized, but no deaths were observed.

**Conclusions:**

The fifth COVID-19 vaccine dose raised antibody response to 93.0% of the study population. Additional booster doses, as well as bivalent vaccines, led to higher levels of antibody concentration in SOT recipients. We found a lower incidence of breakthrough infections among SOT recipients after receiving a bivalent vaccine as a fifth dose compared to those receiving a monovalent dose. Antibody concentrations did not wane when the time between doses was prolonged from four to eight months.

## Introduction

Immunocompromised patients are at increased risk of severe COVID-19. The efficacy of the currently used vaccines against Severe Acute Respiratory Syndrome Coronavirus 2 (SARS-CoV-2) is reduced in recipients of solid organ transplant (SOT). Studies have shown that SOT recipients have the lowest rates of antibody seroconversion after the two first doses of vaccines among immunosuppressed groups (1). Antibody response has been notably assessed by IgG response against the S1-subunit of the spike protein in SARS-CoV-2. A meta-analysis based on 44 studies, including 6158 SOT recipients, showed an improvement in antibody response rate after a third dose to 66% compared to 34.2% after two doses. The meta-analysis, however, also highlighted a challenge in this patient group, as one-third of the test subjects did not develop any antibody response at all (2).

The improvement in antibody response in SOT recipients has been investigated after a fourth and fifth dose. Despite a limited number of participants, these studies showed that the proportion of humoral responses in SOT recipients increased with the number of doses of the monovalent SARS-CoV-2 vaccine (3–6). This was also the conclusion in a larger retrospective study of kidney transplant recipients where the cumulated response rate was 88.7% after five vaccine doses (7).

The evolution of the SARS-CoV-2 variants has reduced the effectiveness of the first version of the COVID-19 vaccine; hence, newer Omicron-adapted bivalent vaccines have been developed (8). The bivalent vaccines either contain two mRNAs encoding: 1) ancestral SARS-CoV-2 (Wuhan-Hu-1) and the BA.1 Omicron variant spike protein or 2) ancestral SARS-CoV-2 (Wuhan-Hu-1) and Omicron BA.4/BA.5 spike protein. Previous studies demonstrated that the bivalent vaccines with BA.1 strain resulted in higher levels of neutralizing antibody response against omicron BA.1 in immunocompetent adults than with the earlier monovalent mRNA vaccines, with a geometric mean titer of neutralizing antibody response against BA.1 at 2372.4 after a bivalent vaccine compared to 1473.5 after the monovalent vaccine (9). Another study found that immunocompromised individuals who received a bivalent booster with BA.4/BA.5 strains had strong neutralizing titers against BA.4/BA.5 compared to those who received monovalent vaccines, but the response to XBB.1.5 and BQ.1.1 was almost the same as the monovalent group (10). However, data from SOT recipients receiving bivalent vaccines against SARS-CoV-2 and knowledge of the overall impact on the humoral immune response of continuous booster doses over time is limited.

The COVAC-Tx study is a prospective study investigating the humoral response after vaccination against SARS-CoV-2 infection in SOT recipients (lung, heart, liver, and kidney) since January 2021. We reported an increased proportion of humoral response from 49% to 77% following the third vaccine dose (11). Nonetheless, this was lower than the 99% response rate seen in healthy controls after a third dose (11). It is crucial to estimate if further vaccine doses and the newest bivalent vaccines affect the humoral immunity in this patient group and protect from the development of severe COVID-19.

The aim of this prospective cohort study was to investigate the spike IgG antibody development against SARS-CoV-2 in adult SOT recipients during the first two years after the first dose of mRNA vaccination. Our specific objectives were to: i) investigate antibody concentrations after each vaccine dose and evaluate the development in antibody concentrations between vaccine doses, ii) investigate the number of breakthrough infections, hospitalizations, need for monoclonal antibody (mAb) treatment, and deaths due to SARS-CoV-2, iii) investigate any potential difference in antibody concentrations, breakthrough infections, hospitalization, and deaths when comparing monovalent, bivalent BA.1 and bivalent BA.4/5 vaccines as a fifth dose.

## Methods

### Population

Adult SOT recipients (≥18 years of age) followed at Odense University Hospital in the Region of Southern Denmark were invited in January 2021 to participate in this study if they had received the first dose of vaccine against SARS-CoV-2. A total of 438 SOT recipients have accepted the invitation and consented to participate in the COVAC-Tx study. The study population is previously described (12) and was followed from January 2021 until March 2023 in this study.

When investigating the proportion of vaccine responders, individuals who had received mAb treatment or had a breakthrough infection were excluded. Breakthrough infection was defined as a positive PCR test or a positive registered rapid test. Comorbidities were identified based on Quan’s coding algorithms for ICD-10 codes in the years 2016-2020 (13). To prevent the inclusion of SOT as comorbidity, relevant SOT recipients were excluded in the following categories: heart disease (myocardial infarction and/or congestive heart failure), chronic pulmonary disease, liver disease (mild and/or moderate/severe liver disease), and renal disease.

### Vaccine type and timing

All participants in the COVAC-Tx study had received mRNA vaccines (BNT162b2 or mRNA-1273) since their first vaccination.

The Danish Health Authority has defined SOT recipients as a prioritized group, and they were offered a third COVID-19 vaccine dose in September 2021, a fourth dose in January/February 2022, and a fifth dose in October/November 2022. The fifth dose is a monovalent or bivalent dose containing mRNAs encoding Wuhan-1 and either Omicron BA.1 or Omicron BA.4/BA.5.

### Data collection

In Denmark, all residents have their own unique social security number, which enables the linking of data across registers<n.o></no> The date and type of vaccination were retrieved from the Danish Vaccination Registry and results of the SARS-CoV-2 PCR test were obtained from the Danish Microbiology Database MiBa (Statens Serum Institut Copenhagen, Denmark). Hospitalizations and treatment regarding SARS-CoV-2 were obtained from the participants’ electronic medical records. The severity of COVID-19 infections was divided into asymptomatic, mild, moderate, severe, or critical according to COVID-19 Treatment Guidelines Panel (14). By using Research Electronic Data Capture (REDCap) hosted by Open Patient Data Explorative Network (15), all data were entered into a secured electronic clinical record.

### Timing of blood sample collection

**Figure 1:**
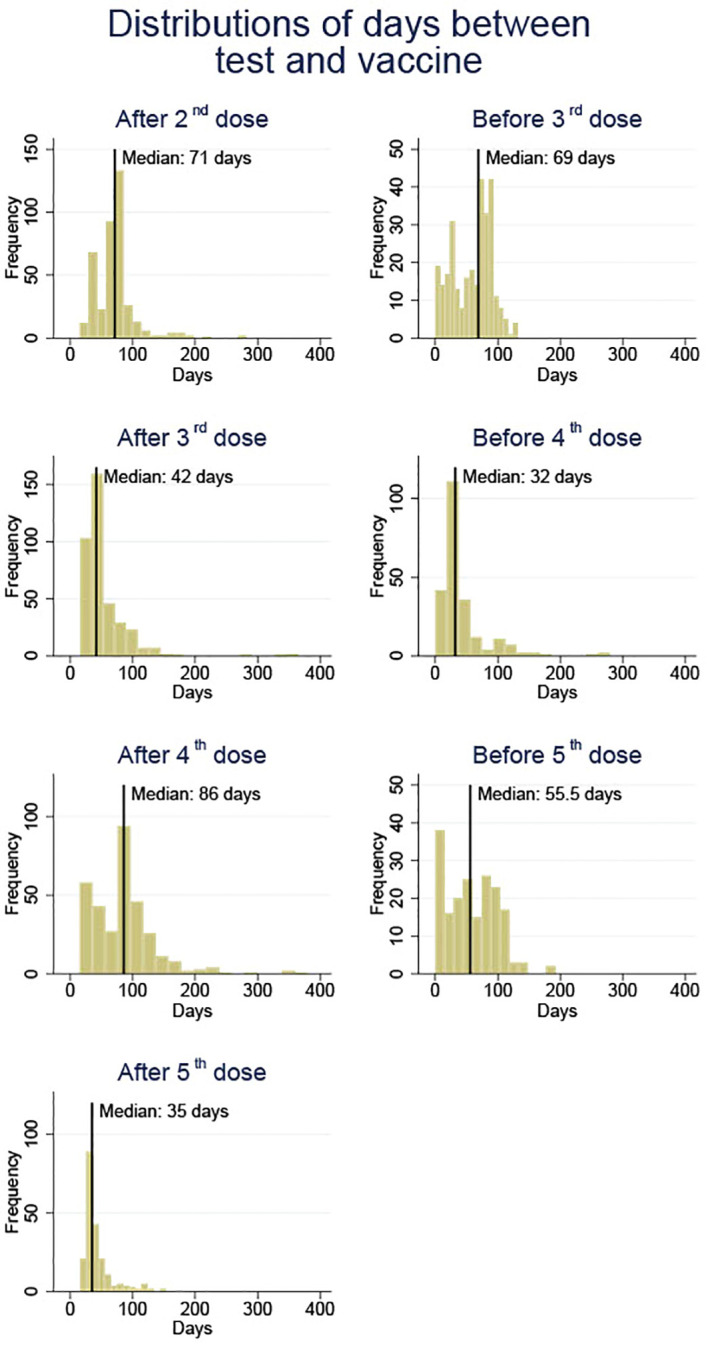
An overview of blood sample collection times before and after each vaccine dose (doses 2 to 5) against SARS-CoV-2 in solid organ transplant recipients.

Blood samples were collected before and after each SARS-CoV-2 vaccine injection since the second dose. **Figure 1** provides an overview of the time when blood samples were obtained as well as the median of the timing. The blood samples before vaccination were taken as late as the hour before vaccination or up to 140 days before the next vaccination. The post-vaccination blood samples were collected between 14 days following the vaccination and a maximum of 381 days prior to the next vaccination. Blood samples were frozen until analysis.

### Laboratory analysis

The Immunogenicity of COVID-<19no></no> vaccination was investigated through analysis of IgG antibodies targeting the receptor-binding domain (RBD) in the S1-subunit of the spike protein in the Wuhan strain of SARS-CoV-2, using the SARS-CoV-2 IgG II Quant Assay (Abbott Laboratories). The relationship between the Abbott arbitrary units (AU)/mL unit and the World Health Organization BAU/mL unit follows the equation BAU/mL = 0.142 × AU/mL (as established by the manufacturer), corresponding to a cut-off of 7.1 BAU/mL. The measuring range for this assay is 7.1 – 5,680 BAU/mL. Results >5,680 BAU/mL are reported as such.

### Statistics

Participants’ characteristics are reported as proportions (%) with median values and quartiles 1 and 3. Standard statistical procedures were used to estimate means with 95% confidence intervals (CI) for log scale transformed antibody concentrations as well as changes in antibody concentrations. However, due to deviation from normal distributions, bootstrapping procedures (using 1000 repetitions) were used to estimate the 95% CI. T-tests were used to test for statistical significance of differences in changes over time of antibody titers. The statistical significance level was set at p < 0.05. Stata 17 was used for all analyses (StataCorp. 2021. *Stata Statistical Software: Release 17*. College Station, TX: StataCorp LLC).

### Ethics

All patients in this study have signed informed consent and could withdraw their consent at any time<n.o></no>

The COVAC-Tx study was conducted in accordance with the Declaration of Helsinki and approved by the Regional Committees on Health Research Ethics for Southern Denmark ID S-20210007C and the Danish Data Protection Agency (j.no 21/8390).

## Results

### Baseline characteristics

**Figure 2:**
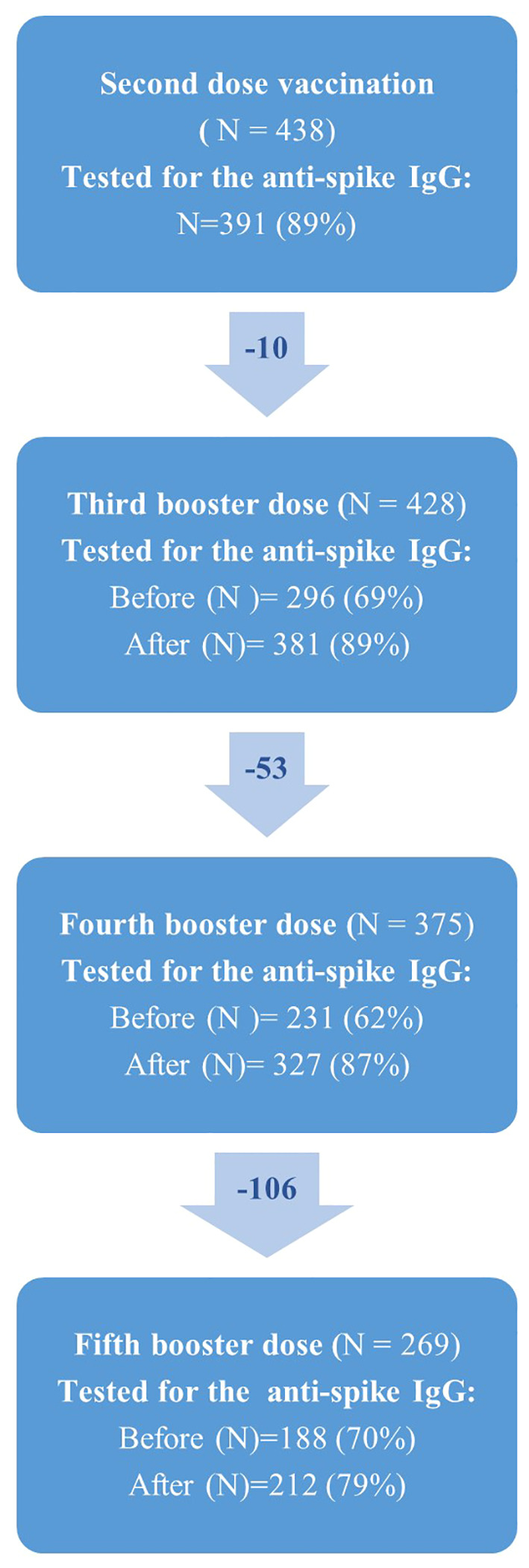
Flowchart showing the number of solid organ transplant recipients (N) who received second, third, fourth and fifth vaccine dose against SARS-CoV-2 in the COVAC-TX project. Due to missing blood samples from participants, N does change over time.

Baseline characteristics of the study population are shown in **Table 1**. In total, 438 SOT recipients participated in the COVAC-Tx study, with a median age of 57.3 years and 59.6% (n=261) were men. Renal and liver diseases together with diabetes mellitus were the most frequent comorbidities. Most participants were kidney transplant recipients (KTRs) (72.6%) receiving proliferation inhibitors and calcineurin inhibitors (**Table 1**). Less than 20% of the participants were transplanted within 2 years and 61.4% received the fifth vaccine dose (**Figure 2**). Of the 438 SOT recipients, all but three received BNT162b2 for all doses. In addition, eight received mRNA-1273 as a fifth dose.

**Table 1:** Baseline demographic and clinical characteristics of solid organ transplant recipients in the study.

Study population, (n=438)	
Age, median (Quartile 1 and Quartile 3) Sex (males), n (%)	57.3 (47.7-66.4) 261 (59.6%)
Comorbidities, n (%)	
Diabetes mellitus Malignancy Peripheral vascular disease Chronic pulmonary disease^a^ Heart disease^b^ Renal disease^c^ Cerebrovascular disease Rheumatic disease Liver disease^d^	87 (19.9%) 29 (6.6%) 26 (5.9%) 21 (6.9%) 21 (5.7%) 25 (76.3%) 16 (3.7%) 13 (3.0%) 16 (19.9%)
Type of transplant, n (%)	
Kidney Liver Heart Lung Combined	318 (72.6%) 73 (16.7%) 20 (4.6%) 17 (3.9%) 10 (2.3%)
Immunosuppressive treatment, n (%)	
Prednisolone Calcineurin inhibitors^e^ Proliferation inhibitors^f^ mTOR inhibitors^g^	109 (24.9%) 342 (78.1%) 401 (91.6%) 2 (0.5%)
Time from transplantation, n (%)	
≤ 1 year 1-2 years 2-5 years 5-10 years > 10 years Missing	31 (7.1%) 46 (10.5%) 88 (20.1%) 128 (29.2%) 141 (32.2%) 4 (0.9%)

^a^Lung transplants excluded. ^b^Heart transplants excluded. ^c^Kidney transplants excluded. ^d^Liver transplants excluded. ^e^Calcineurin inhibitor (CNI): tacrolimus and cyclosporine. ^f^Proliferation inhibitors: mycophenolate and azathioprine. ^g^mTOR inhibitors: sirolimus.

**Figure 3:**
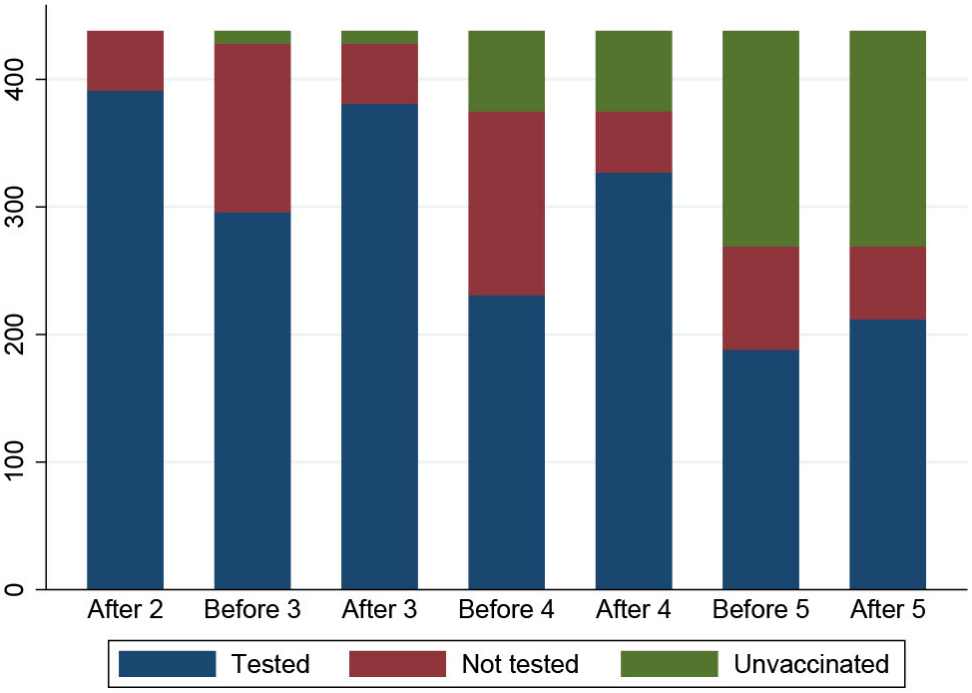
An overview of the number of participants who had a blood sample taken at each time point (N=409). The participants are color coded: blue indicates participants who had a blood sample, red indicates participants who did not have a blood sample and green indicates participants who did not receive booster doses.

**Figure 4:**
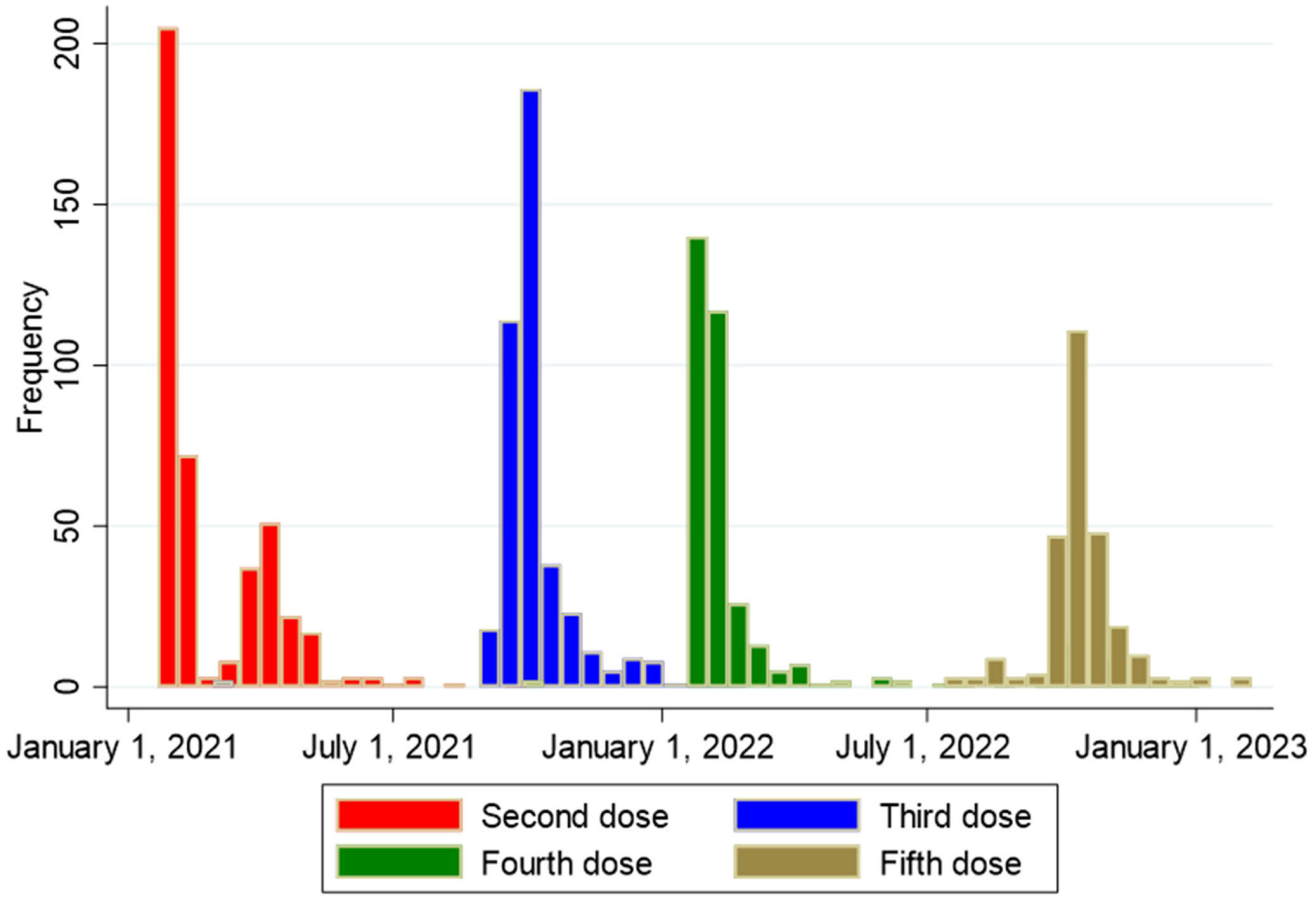
Overview of timing of second, third, fourth and fifth vaccine dose against SARS-CoV-2 among 438 solid organ transplant recipients.

The number of SOT recipients who had antibody concentrations measured after the second dose and before and after each booster dose of the vaccine is shown in **Figures 2**, **3**. The administration and timing of SARS-CoV-2 vaccines and booster doses are illustrated in **Figure 4**. The median timespan between the third and fourth dose was shorter than the time between the fourth and fifth dose; 132 days (Quartile 1 and 3: 123-148) versus 250 days (Quartile 1 and 3: 241-262) (p<0.001).

### Anti-spike IgG concentrations after booster doses

**Figure 5:**
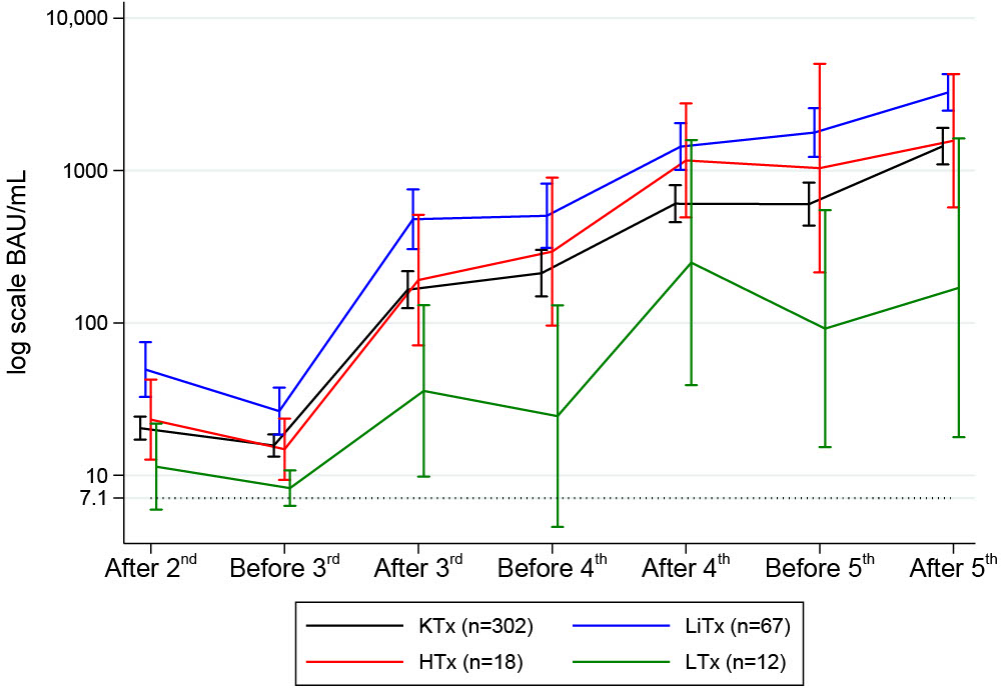
Mean (95%CI) anti-SARS-CoV-2 antibody concentrations to SARS-CoV-2 vaccine before and after second, third, fourth and fifth vaccine dose in solid organ transplant recipients.

Serum anti-spike IgG concentrations are depicted in **Figure 5** and stratified by the transplanted organ. The antibody response among SOT recipients increased after each booster vaccine dose. Following the third dose, spike IgG antibodies were detected in 76.7% (277/361) of the participants. The antibody response increased to 86.7% (164/189) after the fourth dose and 93.0% (93/100) after the fifth dose of the SARS-CoV-2 vaccine. We found no significant differences in age (p=0.76) or sex (p=0.70) among the 7% (7/100) non-responders after the fifth dose. In terms of time since transplantation, the difference among the non-responders varied from 2.2 to 25.6 years. Four were kidney transplanted, one heart transplanted, and two lung transplanted. All but one of the non-responders had one or more of these comorbidities: diabetes, cancer, peripheral vascular disease, and rheumatological disease. We found a higher prevalence of cancers in non-responders (42.9% vs. 8.6%, p=0.03); the remaining comorbidities did not demonstrate any association with vaccine response.

### The development in anti-spike IgG concentrations between vaccine doses

The antibody concentration decreased by 142.7 BAU/mL between the third and the fourth vaccine doses for the uninfected SOT recipients as shown in **Table 2**. Between the fourth and the fifth vaccine dose, this decrease is 234.3 BAU/mL. There is no significant difference (p=0.34) between these decreases in mean antibody concentration among uninfected, despite the difference in time between vaccine doses.

**Table 2:** Mean change in anti-SARS-CoV-2 antibody concentrations following SARS-CoV-2 vaccine in solid organ transplant recipients over time divided uninfected or breakthrough infection, based on infection status between two blood samples, among participants who were uninfected prior to the first of the two sampling points.

	Observations(n)	Mean change in antibody concentration (BAU/mL)	95% confidence interval	P-value
A. from after 3^rd^ dose to before 4^th^ dose				
Uninfected Breakthrough Total Difference	185 39 224	-142.7 1286.6 106.2 -1429.3	[-252.8 -32.6] [833.5 1739.8] [-32.1 244.5] [-1894.6 -964.0]	<0.0001
B. from after 3^rd^ dose to after 4^th^ dose				
Uninfected Breakthrough Total Difference	185 116 301	550.3 2342.1 1240.8 -1791.8	[341.0 759.6] [2000.5 2683.7] [1032.8 1448.8] [-2191.0 -1392.5]	<0.0001
C. from after 4^th^ dose to before 5^th^ dose				
Uninfected Breakthrough Total Difference	96 19 115	-234.3 1622.3 72.4 -1856.6	[-457.7 -11.0] [636.0 2608.7] [-198.7 343.5] [-2863.3 -850.0]	0.001
D. from after 4^th^ dose to after 5^th^ dose				
Uninfected Breakthrough Total Difference	96 19 115	1141.7 2986.4 1551.7 -1844.7	[784.5 1499.0] [2318.4 3654.5] [1212.6 1890.7] [-2595.0 -1094.3]	<0.0001

Only patients who had a blood sample result at both time points were included in the analyses: A: from after the 3^rd^ dose and before the 4^th^ dose, B: from after 3^rd^ dose to after 4^th^ dose, C: from after 4^th^ dose to before 5^th^ dose, D: from after 4^th^ dose to after 5^th^ dose.

### Breakthrough infections

When stratifying the study population by breakthrough infection, the mean change in antibody concentrations following the third vaccine dose until after the fourth dose increased by 550.3<no></no> BAU/mL (95%CI 341.0; 759.6) in uninfected SOT recipients compared to 2342.1 BAU/mL (95%CI 2000.5; 2683.7) in the infected group. The mean change in antibody concentrations after a fourth dose to after a fifth dose was 1141.7 BAU/mL (95%CI 784.5; 1499.0) for the uninfected and 2986.4 BAU/mL (95%CI 2318.4; 3654.4) for the infected participants (**Table 2**).

**Figure 6:**
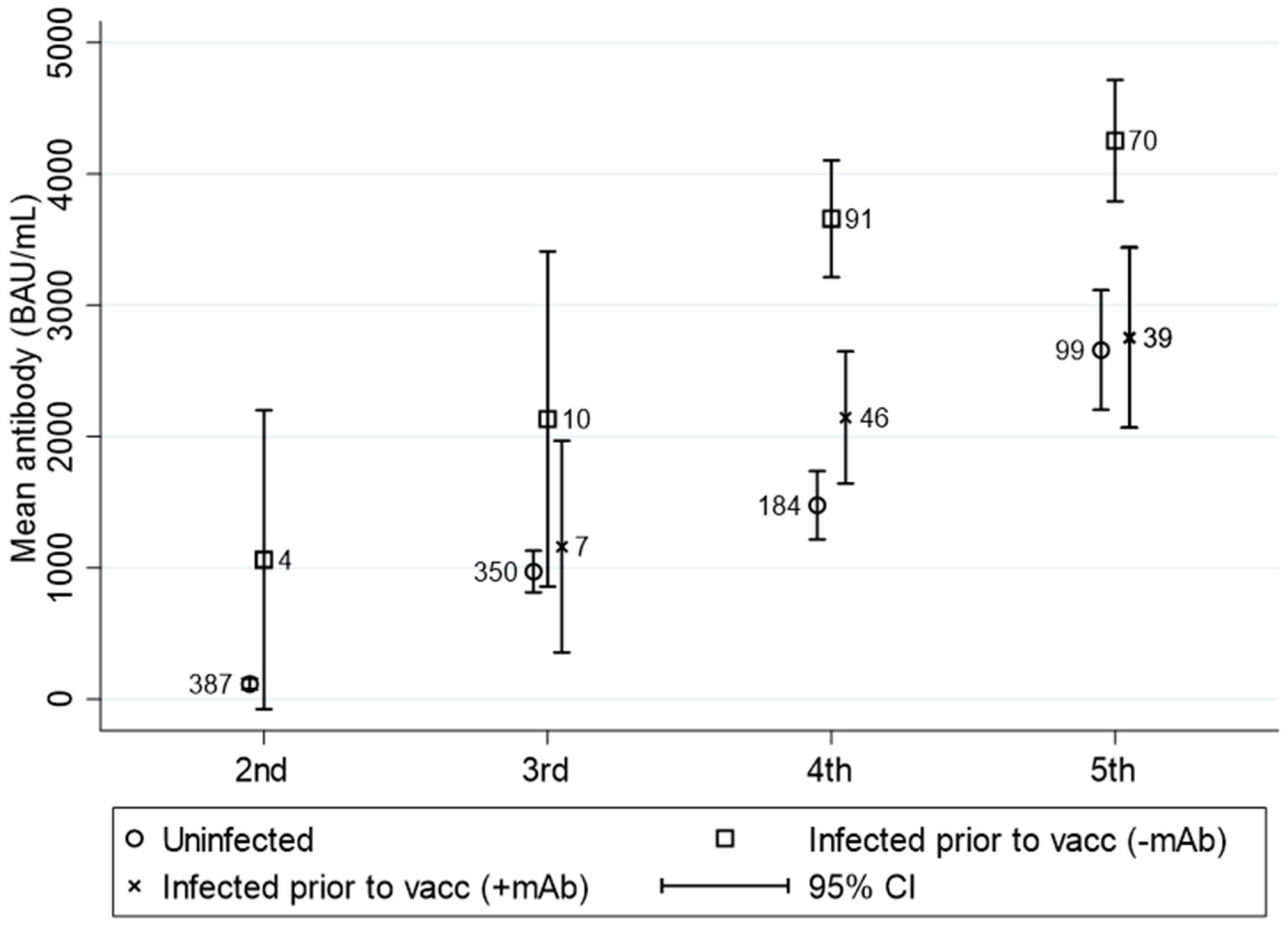
Mean (95%CI) anti-SARS-CoV-2 antibody titers to SARS-CoV-2 vaccine in solid organ transplant recipients divided into uninfected, breakthrough infected prior to vaccination without monoclonal antibody treatment and breakthrough infected prior to vaccination treated with monoclonal antibodies. N is visualized in the numbers next to the dots. This figure includes all patients who were ever infected prior to the testing time.

During the study period, 57.1% (240/420) of the study population had SARS-CoV-2 breakthrough infection at least once. A total of 90 (37.5%) were treated with mAb for mild-moderate COVID-19 infection. The change in antibody concentrations in the vaccinated study population stratified by breakthrough infection is illustrated in **Figure 6**. The antibody concentration increased significantly between the fourth and fifth vaccine doses. The antibody levels following the fourth and fifth vaccinations were significantly higher among those with breakthrough infection without mAb treatment than with no breakthrough infection (p<0.01).

The overall mean antibody concentration two years after the first vaccination against SARS-CoV-2, regardless of the number of vaccine doses, is 48.1% higher in SOT recipients with a breakthrough infection without mAb treatment compared to the uninfected. For the infected SOT recipients that have received mAb treatment, the mean antibody concentration was 12% lower than for the uninfected SOT recipients.

During the study period, 22/240 (9.2%) of SOT recipients with a breakthrough infection were hospitalized. Categorizing the severity of SARS-CoV-2 among the hospitalized 6/22 were mild, 4/22 were moderate, 11/22 were severe, and 1/22 was critical. Of the hospitalized participants, seven had a measured antibody response before hospitalization without any vaccine dose in the time between blood sample and hospitalization. Five patients had no detectable antibody concentration and two had below 1000 BAU/mL. A total of 16/438 (3.6%) died in the study period, but none of these deaths were caused by COVID-19 infection.

### Bivalent vaccine as a fifth vaccine dose

**Figure 7:**
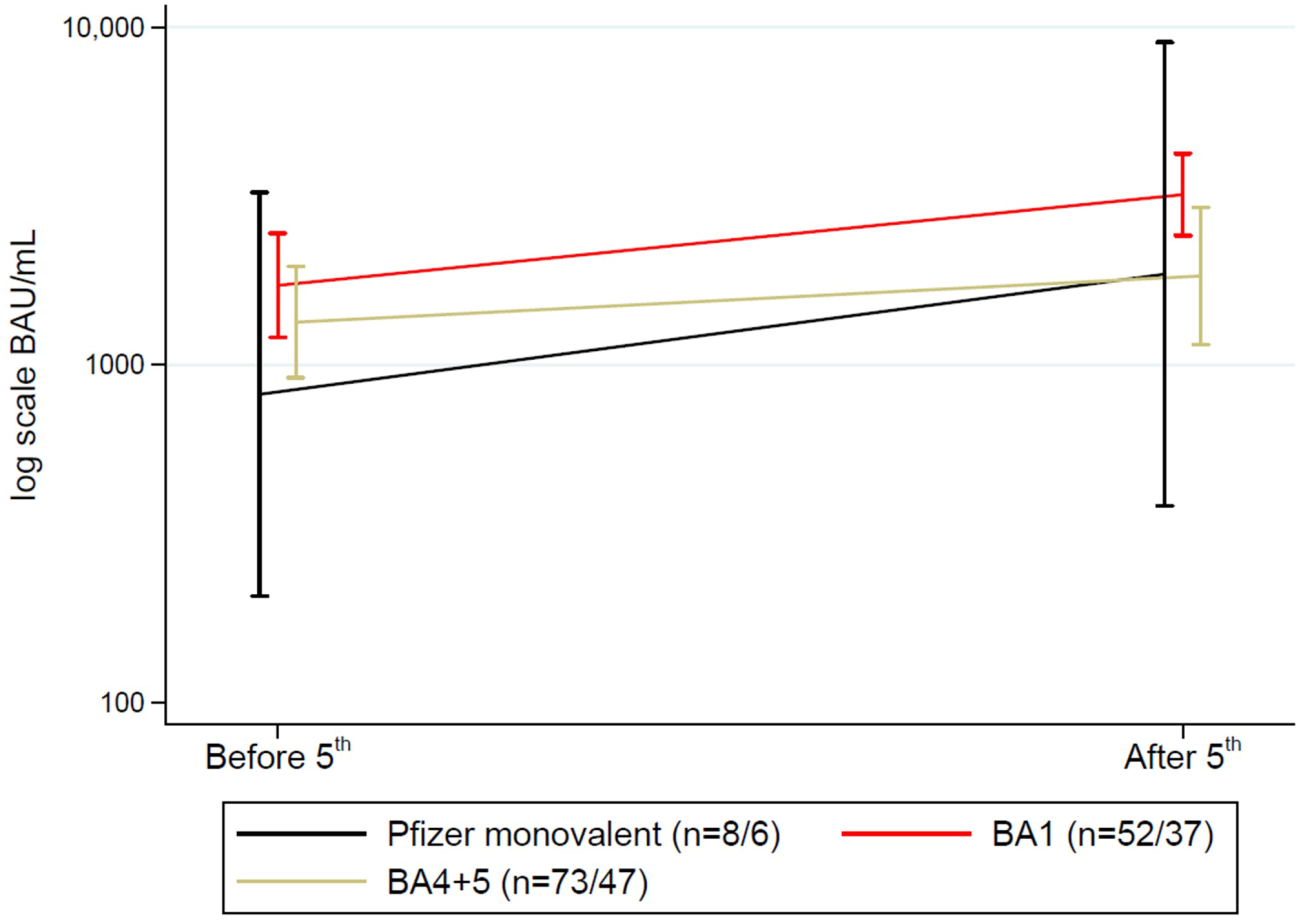
Mean (95%CI) anti-SARS-CoV-2 antibody concentrations to SARS-CoV-2 vaccine before and after a fifth vaccine dose in solid organ transplant recipients divided into variants of vaccines. N shown as (n before 5^th^ dose/n after 5^th^ dose).

Overall, 269<no></no> SOT recipients received a fifth dose, with a bivalent vaccine in 249 participants. Of these, 133 had blood samples taken after a fifth dose, of whom 90 had blood samples before and after the vaccine administration. When comparing the Omicron BA.1 adapted or Omicron BA.4/BA.5 adapted the mean change in antibody concentrations was similar and varied with only 103.45 BAU/mL (p=0.82) (**Figure 7**).

Among those receiving a fifth dose, 20 received a monovalent vaccine, 108 a BA.1 adapted vaccine, and 141 the BA.4/BA.5 adapted vaccine. Nineteen (7.1%) SOT recipients had a breakthrough infection after the fifth dose. There was an association between vaccine type and having a breakthrough infection. Four (20.0%) SOT recipients who received the monovalent vaccine, four participants (4.0%) who received the BA.1 adapted vaccine, and 11 (7.9%) participants who received the BA.4/BA.5 adapted vaccine were infected (p=0.04).

## Discussion

This prospective cohort study found that following each booster vaccine, the antibody concentration against SARS-CoV-2 increased in SOT recipients. After the fifth vaccine dose, 93.0% of SOT-vaccinated recipients had a measurable spike in IgG antibodies. A period of 250 days between vaccine administrations had no significant impact on the decline in antibody concentration compared to 132 days. In addition, we found significantly higher antibody concentrations in vaccinated SOT recipients with breakthrough infections compared to vaccinated without infection. We found an association between the bivalent vaccines as a fifth dose and a lower prevalence of breakthrough infections than breakthrough infections after a fifth, monovalent vaccine dose. The hospital admissions due to breakthrough infection since the administration of the second dose of vaccine were high, and 50% experienced a severe course of COVID-19; however, no deaths were observed.

The increase in humoral response rate after receiving booster doses is in agreement with data from other studies (3, 4, 6, 16). In addition, this study also showed that repetitive booster doses led to a higher percentage of responders, consequently after the fifth dose, 93.0% had developed antibodies against SARS-CoV-2. This tendency is also demonstrated in other studies (1, 4, 5, 17). After four doses, 25 were non-responders and after five doses, only seven individuals were non-responders in this project. This corresponds to Perrier et al. (3) who found that after a fourth dose, 23.8% of the SOT recipients were seronegative and there was no significant correlation to immunosuppressive treatment such as Belatacept in relation to an antibody response. When looking at the causes of the seven seronegative SOT recipients in our study, it was not possible to conclude why these seven individuals did not develop antibodies.

Because of our long follow-up period, it is possible to investigate the timing of vaccine boosters. Both in the general population (18, 19) and in SOT recipients (11), there is a tendency for the antibody concentrations to decrease over time. However, our study showed that despite the longer time (approximately 120 days) between vaccinations, no significant differences in the decrease in antibody concentration were observed. This observation gives a larger window of opportunity to vaccinate SOT recipients without risking a significant decrease in antibody concentration. However, antibody levels are not the only factor to consider when assessing the need for a booster. Still, there was an increase in antibody concentrations after continuous booster doses and, therefore, a beneficial effect of booster vaccines in humoral immunity.

This study population`s hospitalization rate due to COVID-19 over two years was 9.2%. In the general population, only 0.2% of the individuals with breakthrough infections were hospitalized in Denmark (20). Therefore, despite the increase in antibody levels, SOT recipients were still at higher risk of hospitalization due to a breakthrough infection than the general population. However, we found that 10/22 individuals experienced a mild or moderate course of COVID-19 and that no deaths occurred in the hospitalized group. Therefore, the higher hospitalization rate could be due to a more cautious approach to this vulnerable group, even in milder cases. Nevertheless, 11 experienced severe infections and one critical, making the need for hospitalization still greater than in the general population. When only investigating SOT recipients who did not receive a vaccine dose between the latest blood sample and hospitalization, the antibody concentration was not detectable in 5/7 cases, which could suggest that hospitalizations were more frequently seen among non-responders. However, larger studies are needed to clarify the impact of humoral and cellular antibody concentration on the severity of breakthrough infections.

Until now, the studies investigating bivalent vaccines have primarily focused on their effect in the general population (9, 21). These studies have shown that when comparing the mRNA monovalent vaccine to the bivalent BA.5, there is a higher neutralization of Omicron BA.4/BA.5 in individuals receiving the BA.5 booster than the parental booster (21). Another study has recently investigated antibody levels in nursing home residents after receiving a bivalent Omicron BA.4/BA.5 vaccine (22). This study found an increase in anti-spike and neutralizing antibody titers against both Omicron BA.1 and BA.4/BA.5, concluding that the bivalent booster containing Omicron BA.4/BA.5 provides additional protection against SARS-CoV-2 in this group.

Our study provides knowledge regarding the comparison of antibody development after administration of the two different variants of the mRNA-based vaccines (BA.1 or BA.4/BA.5). We found no significant difference in antibody concentrations in SOT recipients receiving their fifth dose as a bivalent Omicron BA.1 containing vaccine and a bivalent Omicron BA.4/BA.5 adapted vaccine. However, the general increase in antibody concentrations was found to be the case in both groups. This finding is in concordance with the ENFORCE study that follows over 6,000 both immunocompetent and immunocompromised Danish individuals. The study likewise observed no difference between antibody concentrations in 2812 individuals who received their fourth dose as a vaccine containing either BA.1 or BA.4/BA.5 (23).

We did not investigate the neutralizing capacities in the whole cohort; however, this was investigated for a subpopulation in this cohort (24). KTRs who were vaccinated with the bivalent mRNA vaccine as a fifth dose, either BA.1 or BA.4/BA.5 displayed a much higher neutralization of the XBB.1.5 Omicron SARS-CoV-2 sub-variant compared to younger healthy individuals and KTRs after the three monovalent vaccine doses (24). This could suggest that humoral antibody response cannot be used as a single method to evaluate the vaccine efficacy.

However, another question arises when comparing antibody concentrations with the corresponding protection against breakthrough infections. The ENFORCE study in Denmark showed an association between higher concentrations of anti-spike antibodies and reduced risk of breakthrough infection with the Delta variant when vaccinated with the Wuhan-Hu-1 SARS-CoV-2 vaccine (20). The same association has not been found with the Wuhan-Hu-1 SARS-CoV-2 vaccine and infections with Omicron variants (20). Other studies demonstrated that after three vaccine doses, there was an association between low antibody concentrations and increased risk of breakthrough infections (25). A study investigated the binding antibody levels in healthcare workers with breakthrough infections (26). The authors found that below 6000 BAU/mL, there was no protection against BA.1. In our study, it was not possible to investigate the correlation between breakthrough infection and levels of antibodies due to very different time points in blood samples, but we found no significant difference in the number of breakthrough infections between the groups receiving the BA.1 adapted vaccine and the BA.4/BA.5 group. However, we found that 4/20 (20.0%) receiving the monovalent vaccine had a breakthrough infection after their fifth dose compared to 4/97 (4.1%) and 11/140 (7.9%) among participants receiving BA.1 and BA.4/BA.5 vaccine, respectively (p=0.04). These findings suggest that there is some protection against breakthrough infections with the current Omicron variants in the group receiving the bivalent vaccines compared to those with monovalent.

## Limitations

This study has some limitations. Firstly, it can be challenging to compare antibody concentrations against SARS-CoV-2 with the risk of breakthrough infection and the severity of these, as it is difficult to claim if this is due to Omicron variants being less severe (27) or if it is due to vaccination status. Secondly, we did not investigate the cellular response or neutralizing antibodies, nor the non-neutralizing functions of antibodies. In addition, the performance of the SARS-CoV-2 IgG II Quant assay, which is developed from the ancestral SARS-CoV-2 (Wuhan-Hu-1), could be questionable when investigating the newer variants of SARS-CoV-2 as measuring the antibodies induced by BA.1 or BA.4/BA.5. The conclusion about the effect of a longer period between the third and fourth vaccine doses compared with the period between the fourth and fifth doses has limitations. In this study, we did not use a mathematical model to reach this conclusion, which could be taken into account when interpreting this result.

We did not investigate the time between vaccination and breakthrough vaccination or the type of monoclonal antibodies in relation to the difference between the level of those who received and did not receive monoclonal antibodies. Many factors can influence this - time, SARS-CoV-2 variant, number of infections, type of monoclonal antibodies.

Our study was a single-center study, which can limit the generalizability especially as the number of lung and heart recipients is quite small compared to the number of kidney and liver transplant recipients. However, this study provides longitudinal data and continuous follow-up. Furthermore, breakthrough infection among the participants might be underestimated. Although SOT recipients are advised to contact their hospital when experiencing symptoms of SARS-CoV-2 or other infections, it is not certain that all adhere to this recommendation. Home testing became more available during the study period and patients with mild symptoms would probably not contact the health care system. This could result in an overestimation of the antibody concentrations in the non-infected group and therefore attributed to the increased effect of the booster doses.

Another limitation of this study is missing data, as antibody data were only accessible from around half of the study population. However, the study population has been followed for two years. With the considerable number of participants in the study period, we demonstrated an antibody response after each booster dose.

## Conclusion

The benefits of repetitive booster doses in SOT recipients induced a clear increase in antibody concentrations. Furthermore, the new bivalent vaccines provide better protection against breakthrough infections than monovalent vaccines. This result indicates the need for continued adjustment of the vaccines to the newest SARS-CoV-2 variants, but also booster doses to SOT recipients, as this population is at higher risk of hospitalization and severe disease course of COVID-19. We observed only a minor reduction in antibody concentrations when comparing four and eight months between vaccine doses, which may be an important issue when planning future vaccination for SOT recipients.

## Data availability statement

The original contributions presented in the study are included in the article/supplementary material. Further inquiries can be directed to the corresponding author.

## Ethics statement

The studies involving humans were approved by Regional Committees on Health Research Ethics for Southern Denmark ID S-20210007C. The Danish Data Protection agency (j.no 21/8390). The studies were conducted in accordance with the local legislation and institutional requirements. The participants provided their written informed consent to participate in this study.

## Author contributions

EC: Writing – original draft, Writing – review & editing. AN: Writing – review & editing, Conceptualization, Data curation. IP: Writing – review & editing, Formal Analysis. SL: Writing – review & editing, Project administration. JD: Writing – review & editing, Conceptualization, Data curation. RA: Writing – review & editing, Conceptualization, Data curation. MP: Writing – review & editing, Conceptualization, Data curation. RP: Writing – review & editing, Conceptualization, Data curation. UJ: Writing – review & editing, Conceptualization. NJ: Writing – review & editing, Project administration. CB: Writing – review & editing, Conceptualization, Data curation. LM: Writing – review & editing, Writing – original draft. IJ: Writing – review & editing, Conceptualization, Project administration, Writing – original draft.
